# Vaginal microbiome knowledge and hygiene practices among women in Saudi Arabia: a cross-sectional study

**DOI:** 10.1186/s12889-025-26075-9

**Published:** 2025-12-28

**Authors:** Sarah Almuhayya, Faye Aldehalan, Lama Alzamil, Karem Ibrahem, Sarah R. Alharbi, Esraa Aldawood

**Affiliations:** 1 Department of Clinical Laboratories Sciences, College of Applied Medical Sciences, King Saud University, Kingdom of Saudi Arabia, Riyadh, 12372 Saudi Arabia; 2 Chair of Medical and Molecular Genetics Research, Department of Clinical Laboratory Science, College of Applied Medical Science, King Saud University, Kingdom of Saudia Arabia, Riyadh, 12372 Saudi Arabia; 3 Department of Clinical Laboratory Sciences, College of Applied Medical Sciences Imam Abdulrahman Bin Faisal University, Dammam, Kingdom of Saudi Arabia; 4 Department of Clinical Microbiology and Immunology, Faculty of Medicine, King Abdulaziz University, Jeddah, Saudi Arabia

**Keywords:** Vaginal microbiome, Feminine hygiene, Lactobacillus, Women’s health, Saudi arabia, Douching, Microbiome awareness, Public health education

## Abstract

**Background and objectives:**

The vaginal microbiome plays a critical role in reproductive health through Lactobacillus-dominated communities that maintain acidic pH and suppress pathogens. Disruptive hygiene practices, such as douching and certain feminine products, may negatively affect this balance. Limited data exist on vaginal microbiome awareness and related behaviors among women in Saudi Arabia. This study aimed to assess women’s knowledge of the human and vaginal microbiome and examine associated hygiene practices.

**Materials and methods:**

A cross-sectional online survey was conducted between November 2024 and February 2025 among women aged ≥ 18 years residing in Saudi Arabia. A total of *N* = 1,040 participants were recruited using convenience and snowball sampling through social media platforms. The questionnaire assessed sociodemographic characteristics, microbiome knowledge, and vaginal hygiene practices. Associations were examined using multivariable linear regression, and results are reported with 95% confidence intervals (CIs).

**Results:**

Participants demonstrated moderate general microbiome knowledge but limited understanding of the vaginal microbiome. Only 20.67% correctly identified *Lactobacillus* as the dominant genus in a healthy vaginal ecosystem. Higher knowledge scores were observed among women with higher education and those working in healthcare. Use of internal vaginal washes was significantly associated with lower vaginal microbiome knowledge (β = − 0.30, 95% CI: − 0.58 to − 0.01, *p* = 0.0403). Other feminine product use showed no significant associations with knowledge levels.

**Conclusions:**

Substantial gaps exist in vaginal microbiome awareness among Saudi women, accompanied by frequent use of potentially disruptive hygiene practices. Targeted health education and culturally appropriate interventions are needed to promote microbiota-supportive behaviors and improve reproductive health outcomes.

**Supplementary Information:**

The online version contains supplementary material available at 10.1186/s12889-025-26075-9.

## Introduction

The human vaginal microbiome plays a vital role in preserving female reproductive health [1]. It consists of a diverse array of microorganisms, predominantly bacteria, that coexist within the vaginal ecosystem [2]. A healthy vaginal microbiome is typically dominated by *Lactobacillus* species, which are widely recognized as the core organisms of the vaginal ecosystem in reproductive-age women [3]. Culture-independent studies have identified five major community state types of the vaginal microbiota, three to four of which are characterized by a predominance of *Lactobacillus* species, often comprising more than 90% of the microbial population [4, 5]. These bacteria contribute to vaginal health through multiple protective mechanisms. They help maintain an acidic environment (pH < 4.5) [6], primarily by producing lactic acid and hydrogen peroxide [7]. Additionally, *Lactobacilli* synthesize a variety of bacteriostatic and bactericidal compounds and inhibit pathogen colonization through competitive exclusion [8, 9]. This hostile environment for pathogenic organisms prevents their overgrowth and has been associated with a reduced risk of infections, including bacterial vaginosis (BV), candidiasis, urinary tract infections (UTIs), and adverse obstetric outcomes such as preterm birth [3, 10].

The vaginal microbiome exhibits considerable interindividual variability, shaped by a multifaceted interplay of biological, behavioral, and environmental determinants. Sexual activity, for instance, has been associated with shifts in microbial composition and reduced dominance of *Lactobacillus* species, which are crucial for maintaining a stable, low-pH vaginal environment [11]. Psychological and chronic stress can modulate immune responses and mucosal integrity, thereby influencing microbial equilibrium [12]. Geographic and regional differences further contribute to distinct microbiota profiles due to variations in climate, lifestyle, and access to healthcare services [13]. Additionally, racial and ethnic background has been linked to specific vaginal community state types, potentially reflecting genetic predispositions and sociocultural influences [1] Other important contributors include hormonal fluctuations, contraceptive use, antibiotic exposure, and systemic health conditions [14].

Disruptions to this microbial balance, known as dysbiosis, have been linked to increases risks of various diseases, including bacterial vaginosis and preterm birth [15, 16]. Long-standing dysbiosis has also been associated with recurrent bacterial vaginosis, increased susceptibility to sexually transmitted infections, chronic inflammation, infertility, and other adverse reproductive outcomes [17–19]. Although antibiotic treatments are effective in eliminating pathogens, recurrence occurs in up to 50% of cases, primarily due to the failure to re-establish a healthy vaginal microbiota [20, 21].

Factors contributing to vaginal dysbiosis are multifaceted and include both internal and external influences. The use of antibiotics disrupts the microbial balance by reducing beneficial Lactobacillus populations, allowing opportunistic pathogens to proliferate [22, 23]. Hormonal changes, particularly fluctuations in estrogen levels during menstruation, pregnancy, or menopause, can significantly alter the vaginal microbial composition [1, 24]. Menstrual hygiene practices, such as prolonged use of tampons or pads, may create an environment conducive to microbial imbalance [23]. Sexual activity introduces foreign microorganisms and alters the vaginal environment, which can disturb microbial stability [11]. Personal hygiene behaviors, including intravaginal douching and the use of scented or antiseptic feminine products, have been repeatedly associated with increased risk of dysbiosis, bacterial vaginosis, and other reproductive health disorders [25, 26].

Notably, vaginal douching has drawn significant scientific attention as a key modifiable risk factor for dysbiosis, with studies showing it directly alters the vaginal microenvironment and predisposes individuals to microbial imbalance and subsequent infections [25]. It involves rinsing the vagina with water or other solutions, including antiseptics and fragrances. Although intended to enhance cleanliness or eliminate odor, douching can disrupt the protective Lactobacillus-dominated microbiota, increase vaginal pH, and facilitate the growth of anaerobic bacteria associated with bacterial vaginosis and other infections [1]. Health authorities, including the CDC, strongly discourage douching due to its association with pelvic inflammatory disease, ectopic pregnancy, and infertility [25].

Beyond douching, women increasingly use a variety of feminine hygiene products such as vaginal sprays, wipes, powders, and washes. These products are often promoted through messaging that reinforces shame about natural secretions, pushing the narrative that normal vaginal physiology requires correction. Many contain alcohol, fragrances, and antimicrobial agents that can irritate the mucosa and disrupt the microbial balance [25, 27]. In addition to these products, intimate grooming practices have become common, yet their potential effects on the vaginal environment are rarely examined. While these practices are widely reported globally, region-specific data, particularly from Saudi Arabia and the broader Middle East, remain limited.

Cultural values surrounding modesty, social taboos, and limited access to open reproductive health discourse can significantly shape women’s hygiene behaviors. These influences often lead to the widespread adoption of practices that are poorly understood in terms of their health implications. For example, vaginal douching and steam therapies are traditionally practiced in many Middle Eastern cultures, particularly after menstruation or childbirth, under the belief that they promote cleanliness and sexual desirability [27]. Similarly, body hair become closely associated with femininity and hygiene in the region, despite limited research on their potential microbiological consequences.

In Saudi Arabia, limited published research has examined these practices in relation to microbiome health. However, emerging research highlights a general lack of knowledge about in microbiome. In a previous study by Alamri and AlKhater (2022), authors found that while awareness of the human microbiome was moderate among students in clinical laboratory sciences, nutrition, and public health, knowledge of dysbiosis, its health implications, and therapeutic interventions such as probiotics and fecal microbiota transplantation was significantly limited. Notably, their study did not explore the vaginal microbiome specifically or link this knowledge to hygiene or grooming behaviors [28].

Furthermore, a comprehensive review highlighted that microbiome research in Saudi Arabia over the past decade has predominantly focused on limited health and environmental aspects, with minimal application to women’s health and the vaginal microbiome. This underscores the need for more applied microbiome research and education initiatives, particularly those addressing the implications of personal hygiene practices on women’s health [29]. Recent regional studies support this call for action. For example, Parveen and his colleagues conducted a nationwide survey assessing oral microbiome knowledge among dental professionals in Saudi Arabia [30]. The study revealed notable gaps in awareness, particularly regarding the clinical relevance of the oral microbiome in disease prevention and health promotion, emphasizing the importance of professional education in microbiome science. Health education interventions in Saudi Arabia have shown promise in improving reproductive health knowledge. For example, a previous study demonstrated that targeted awareness campaigns among university students significantly increased understanding of reproductive health topics [31].

In light of these observations, the current study examines vaginal health practices among women in Saudi Arabia. By assessing the use of feminine hygiene products and douching, this research explores the relationship between intimate care behaviors and microbiome knowledge. This broader perspective supports the development of targeted health education strategies while promoting microbiota-friendly practices to reduce infection-related morbidity and support women’s overall well-being.

## Materials and methods

###  Study design and setting

Data were collected through distributing an online questionnaire via social media platforms. Eligible participants included women aged 18 years and older residing in Saudi Arabia who were able to read Arabic or English. Male respondents and individuals under 18 years were excluded. The required sample size for this study was calculated to be 377 participants using Raosoft, Inc. (Seattle, WA, USA) (http://www.raosoft.com/samplesize.html) (accessed on 26 June 2024), with a 95% confidence level and a 5% margin of error. However, a total of 1,040 women completed the survey and were included in the analysis.

Participants were recruited using a convenience and snowball sampling approach. The survey link was distributed via WhatsApp and Telegram in publicly accessible and interest-specific group chats. Participants were encouraged to share the link within their networks to maximize reach. No targeted advertising or quotas were used. Eligibility was confirmed before survey access. The survey was set to require responses to all questions, thus eliminating incomplete entries. Duplicate responses were checked via timestamp and IP address patterns, and none were identified. Therefore, no responses were excluded due to duplication or incompleteness.

###  Data collection

This cross-sectional study was conducted between November 2024 and February 2025 using a self-administered online questionnaire. A pilot test was conducted prior to the main data collection phase and included 14 women. The purpose of the pilot was to evaluate the clarity, relevance, and estimated time required to complete the survey. The tool underwent face validity testing with expert consultants and adjustments were made based on feedback to improve the final version of the questionnaire. The questionnaire was originally developed in English, translated into Arabic, and then back-translated by a bilingual expert to ensure linguistic accuracy and conceptual equivalence. No substantive differences existed between the two versions, and both were identical in content, structure, and scoring. Items assessing general microbiota knowledge were adapted from previously validated surveys examining microbiome awareness in health sciences students and the general population [28, 32, 33]. The True/False/“I Don’t Know” structure and scoring approach were informed by established HPV knowledge instruments [34, 35]. These sources guided item construction, scoring methodology, and content refinement. Internal consistency was assessed using Cronbach’s alpha. The 8-item general microbiota knowledge score (KS) demonstrated good reliability (α = 0.72), indicating that participants who answered one item correctly tended to answer others correctly. Similarly, the 5-item vaginal microbiota knowledge score (VMK) also showed acceptable internal consistency (α = 0.73). An English version of the final questionnaire is provided as Supplementary File 1.

The final survey instrument was organized into four thematic domains. The first domain collected sociodemographic data, such as age, marital status, educational background, employment status, affiliation with the healthcare sector, and geographic region within Saudi Arabia. The second domain focused on general awareness of the human microbiome, assessing participants’ knowledge about its composition and role in human health. The third domain addressed knowledge of the vaginal microbiome and related hygiene practices, evaluating understanding of the vaginal microbial ecosystem and its implications for women’s health. The final domain examined personal hygiene behaviors and sources of information, particularly the use of vaginal hygiene products and the channels through which participants accessed health-related knowledge.

All knowledge-based questions were developed in consultation with microbiologists and presented in a True/False/I Don’t Know format to assess both awareness and uncertainty. The finalized survey was distributed electronically after receiving ethical approval. To ensure broad geographic representation, data collectors from various regions across the country assisted in disseminating the survey link via digital platforms.

###  Ethical considerations

All procedures performed in this study were conducted in accordance with the ethical standards of the institutional research committee and with the Declaration of Helsinki. Prior to participation, all respondents were informed of the study’s objectives and provided informed, voluntary consent before accessing the questionnaire. Participation was entirely anonymous, and all responses were treated with strict confidentiality. Ethical approval for the study was obtained from the Institutional Review Board at King Saud University (Ref. No. 24/1508/IRB) (22 August 2024). Access to the collected data was restricted exclusively to members of the research team to ensure privacy and data protection.

###  Statistical analysis

Descriptive statistics (frequencies, percentages, means, and standard deviations) were used to summarize participant demographics, microbiome knowledge levels, and hygiene product use. Knowledge on the human microbiome was assessed using Chi-square test of independence to evaluate whether there were significant differences in the distribution of correct and incorrect answers across the different statements. For prediction of vaginal microbiota knowledge by vaginal hygiene practices, linear regression analysis was conducted for each listed practice to evaluate its predictive association with vaginal microbiota knowledge (VMK score, range 0 to 5), both unadjusted and adjusted linear regression analyses were performed. Unadjusted models examined each vaginal hygiene practice individually as an independent predictor of VMK. In addition, a multivariable linear regression model was conducted including all hygiene practices and key sociodemographic covariates (age group, marital status, education, occupation, healthcare affiliation, and region) to assess adjusted associations.

For prediction of general microbiota knowledge (KS score, range 0–8), a multivariable linear regression was used with sociodemographic characteristics entered simultaneously to adjust for potential confounding. Regression coefficients (β), 95% confidence intervals (CIs), and p-values were reported to evaluate the contribution of each predictor. Assumptions of linearity, independence, and homoscedasticity were verified. A p-value < 0.05 was considered statistically significant. All analyses were conducted using GraphPad Prism (version 10.4.1) and Excel (Microsoft 365).

## Results

### Sociodemographic characteristics of participants

A total of 1,040 women from different regions of Saudi Arabia participated in this cross-sectional study (Table 1). The largest age group was 20–30 years (45.96%), followed by equal proportions in the < 20 and 30–40 year groups (17.12% each). A smaller percentage of participants were aged 40–50 years (14.42%) and over 51 years (5.38%). Most participants were single (58.36%), while 36.15% were married, 4.13% divorced, and 1.35% widowed. In terms of education, the majority held a bachelor’s degree (68.56%), followed by high school graduates (21.73%), postgraduates (8.17%), and those with only elementary education (1.54%). Regarding employment, 45.29% of participants were students, 24.23% were employed full-time, 3.27% part-time, and 4.52% were self-employed. Additionally, 19.03% were unemployed and 3.65% were retired. Notably, 32.79% of the sample reported working in the healthcare sector. Geographically, the majority of respondents were from the Central region (71.73%), while the remainder were from the Northern (9.14%), Eastern (7.88%), Western (6.73%), and Southern (4.52%) regions.

**Table 1:** Demographic characteristics of the participants (*N* = 1040)

Item	*n* (%)
Age	
< 20 years	178 (17.12)
20–30 years	478 (45.96)
30–40 years	178 (17.12)
40–50 years	150 (14.42)
> 51 years	56 (5.38)
Marital Status	
Single	607 (58.36)
Married	376 (36.15)
Divorced	43 (4.13)
Widow	14 (1.35)
Education	
Elementary	16 (1.54)
High school	226 (21.73)
Bachelor	713 (68.56)
Postgraduate	85 (8.17)
Occupation	
student	471 (45.29)
Employed full-time	252 (24.23)
Employed part-time	34 (3.27)
Self employed	47 (4.52)
Unemployed	198 (19.03)
Retired	38 (3.65)
In healthcare	
Yes	341 (32.79)
No	699 (67.21)
Region	
Central	746 (71.73)
Western	70 (6.73)
Eastern	82 (7.88)
Northern	95 (9.14)
Southern	47 (4.52)

###  Participants’ knowledge of the human microbiome

Participants demonstrated varying levels of knowledge regarding the human microbiome (Table 2). While 44.33% correctly identified the microbiome as the collection of all microorganisms residing in the human body, 55.67% either misunderstood or were unfamiliar with the term. A majority (85.38%) incorrectly believed that all microorganisms on the human body are harmful, reflecting a common misconception about the role of beneficial microbes. Most participants were aware of microbial presence in the intestinal tract (83.65%) and on the skin (71.44%). Awareness was lower for the respiratory tract (49.52%) and moderate for the vaginal area (71.83%). Additionally, only 47.60% recognized that microbiome composition varies between individuals. Notably, 75.19% of respondents acknowledged that antibiotic misuse can negatively affect the human microbiota, indicating some awareness of the microbiome’s sensitivity to external factors.

**Table 2:** Participants’ knowledge of the human Microbiome (*N* = 1040)

	Correct *n* (%)	Incorrect *n* (%)
Statements		
The term ‘Microbiome’ refers to all microorganisms in the human body.	461 (44.33)	579 (55.67)
All microorganisms found on the human body are harmful.	888 (85.38)	152 (14.62)
Microbiome composition is similar for all people.	495 (47.60)	545 (52.40)
There are microorganisms living naturally in the intestinal tract?	870 (83.65)	170 (16.35)
There are microorganisms living naturally in the respiratory tract?	515 (49.52)	525 (50.48)
There are microorganisms living naturally on the skin?	743 (71.44)	297 (28.56)
There are microorganisms living naturally in the vagina.	747 (71.83)	293 (28.17)
Antibiotics misuse can negatively impact the microbiota	782 (75.19)	258 (24.81)

### Association between sociodemographic factors and human microbiome knowledge

Multivariable linear regression analysis identified several sociodemographic factors significantly associated with participants’ knowledge of the human microbiome (Table 3). Participants aged 30–40 years demonstrated significantly higher knowledge scores compared to those aged 20–30 years (β = 0.54, 95% CI: 0.04 to 1.03, *p* = 0.0334). Educational level was also a significant predictor; individuals with only a high school education had lower knowledge scores than those with a bachelor’s degree (β = − 0.46, 95% CI: − 0.78 to − 0.13, *p* = 0.0065). Employment in the healthcare sector was the strongest positive predictor of microbiome knowledge (β = 1.36, 95% CI: 1.06 to 1.66, *p* < 0.0001). Other variables, including marital status, occupational category, and geographic region, were not significantly associated with microbiome knowledge in the adjusted model.

**Table 3:** Multivariable linear regression predicting general microbiota knowledge by sociodemographic characteristics (*N* = 1040)

Sociodemographic characteristics	Knowledge on the Human Microbiome		*p* value
	Beta Estimate	95% CI	
Age			
*< 20 years*	−0.08	−0.46 to 0.30	0.6751
*20–30 years*	*Ref*		
*30–40 years*	0.54	0.04 to 1.03	0.0334 *
*40–50 years*	0.50	−0.03 to 1.03	0.063
*> 51 years*	0.31	−0.54 to 1.15	0.4733
Marital Status			
*Single*	*Ref*		
*Married*	−0.26	−0.71 to 0.19	0.2683
*Divorced*	−0.53	−1.27 to 0.20	0.1563
*Widow*	−0.48	−1.73 to 0.77	0.4557
Education			
*Elementry*	−0.77	−1.93 to 0.38	0.1899
*High school*	−0.46	−0.78 to −0.13	0.0065 **
*Bachelor*	Ref		
*Postgraduate*	−0.18	−0.69 to 0.32	0.4744
Occupation			
*student*	*Ref*		
*Employed full-time*	−0.11	−0.57 to 0.35	0.6321
*Employed part-time*	−0.49	−1.24to 0.27	0.2077
*Self employed*	−0.15	−0.83 to 0.54	0.6742
*Unemployed*	0.08	−0.39 to 0.55	0.7503
*Retired*	0.53	−0.41 to 1.47	0.2686
In healthcare			
*Yes*	1.36	1.06 to 1.66	< 0.0001 ****
*No*	*Ref*		
Region			
*Central*	*Ref*		
*Western*	0.31	−0.19 to 0.82	0.2277
*Eastern*	0.03	−0.45 to 0.50	0.909
*Northern*	0.43	−0.03 to 0.89	0.0665
*Southern*	−0.12	−0.73 to 0.49	0.706

### Participants’ knowledge of the vaginal microbiome

Participants exhibited varied levels of knowledge regarding the vaginal microbiome (Table 4). A majority (62.40%) correctly recognized that the vaginal microbiome plays an essential role in maintaining vaginal health and protecting against pathogenic microorganisms. Additionally, 60.29% were aware that antibiotic use can cause temporary changes in the vaginal microbial community, and 56.73% acknowledged that douching can disrupt the vaginal microbiota. Fewer participants were aware of more specific aspects of the vaginal microbiome. Only 48.65% correctly indicated that the composition of the vaginal microbiota can change throughout the menstrual cycle, and 20.67% accurately identified Lactobacillus species as the dominant bacteria in a healthy vaginal environment.

**Table 4:** Participants’ knowledge of the vaginal Microbiome (*N* = 1040)

	Correct *n* (%)	Incorrect *n* (%)
Statements		
The vaginal microbiome plays a crucial role in maintaining vaginal health and defending against pathogenic microorganism.	649 (62.40)	391 (37.60)
Vaginal microbiome is dynamic, dominated by Lactobacillus in health.	215 (20.67)	825 (79.33)
The composition of the vaginal microbiota can change throughout a woman’s menstrual cycle.	506 (48.65)	534 (51.35)
Usage of antibiotics can cause a temporal change in human vaginal microbiota.	627 (60.29)	413 (39.71)
Douching can disrupt vaginal microbiota by altering microbial community.	590 (56.73)	450 (43.27)

### Association between sociodemographic factors and vaginal microbiome knowledge

Multivariable linear regression analysis identified several sociodemographic factors significantly associated with participants’ knowledge of the vaginal microbiome (Table 5). Widowed participants had significantly lower knowledge scores compared to single participants (β = − 1.06, 95% CI: − 2.03 to − 0.09, *p* = 0.0315). Similarly, participants with only a high school education had lower scores than those with a bachelor’s degree (β = − 0.27, 95% CI: − 0.52 to − 0.014, *p* = 0.0385). In contrast, retired individuals had significantly higher knowledge scores compared to students (β = 0.82, 95% CI: 0.094 to 1.55, *p* = 0.027). Employment in the healthcare sector was also positively associated with higher knowledge (β = 0.79, 95% CI: 0.56 to 1.03, *p* < 0.0001). Regionally, participants from the Northern region had significantly higher knowledge scores than those from the Central region (β = 0.60, 95% CI: 0.25 to 0.96, *p* = 0.0009). No significant associations were observed for other variables, including age groups, marital status (excluding widowed), education levels (elementary and postgraduate), most occupational categories, or other geographic regions (Western, Eastern, Southern).

**Table 5:** Multivariable linear regression predicting vaginal microbiota knowledge by sociodemographic characteristics (*N* = 1040)

Sociodemographic characteristics	Knowledge on the Vaginal Microbiome		*p* value
	Beta Estimate	95% CI	
Age			
*< 20 years*	−0.02	−0.31 to 0.28	0.9075
*20–30 years*	*Ref*		
*30–40 years*	0.35	−0.035 to 0.73	0.0751
*40–50 years*	0.26	−0.15to 0.67	0.2093
*> 51 years*	−0.32	−0.98 to 0.33	0.3323
Marital Status			
*Single*	*Ref*		
*Married*	−0.08	−0.43 to 0.27	0.6414
*Divorced*	−0.17	−0.74 to 0.39	0.5559
*Widow*	−1.06	−2.03 to −0.09	**0.0315** *
Education			
*Elementry*	0.63	−0.27 to 1.52	0.1687
*High school*	−0.27	−0.52 to −0.014	**0.0385** *
*Bachelor*	*Ref*		
*Postgraduate*	0.098	−0.29 to 0.49	0.6237
Occupation			
*student*	*Ref*		
*Employed full-time*	−0.07	−0.43 to 0.29	0.699
*Employed part-time*	−0.32	−0.91 to 0.26	0.2819
*Self employed*	−0.12	−0.65 to 0.41	0.6627
*Unemployed*	−0.002	−0.37 to 0.36	0.9915
*Retired*	0.82	0.094 to 1.55	0.027
In healthcare			
*Yes*	0.79	0.56to 1.03	**< 0.0001** ****
*No*	*Ref*		
Region			
*Central*	*Ref*		
*Western*	0.35	−0.050to 0.74	0.0867
*Eastern*	0.17	−0.19 to 0.54	0.3571
*Northern*	0.6	0.25 to 0.96	**0.0009** ***
*Southern*	−0.1	−0.58 to 0.38	0.6733

### Types and prevalence of vaginal health and hygiene product use

Among the 1,040 participants, the use of vaginal health and hygiene products varied between internal and external applications (Table 6). Internal product use was reported by 55.2% of participants, while external product use was more common, reported by 70.2%. The most frequently used internal products were vaginal washes or cleansers (38.17%), followed by anti-itch creams (17.31%) and vaginal moisturizers or lubricants (15.96%). Less commonly used internal products included vaginal deodorant suppositories (9.81%), vaginal tablets (8.75%), vaginal wipes (8.46%), baby or antiseptic wipes (8.17%), and vaginal sprays (3.65%). For external use, vaginal washes or cleansers were the most reported (43.65%), followed by anti-itch creams (22.02%), hand or body lotion (18.85%), and baby oil (14.71%). Vaginal moisturizers or lubricants (13.56%), shaving cream (13.56%), and vaginal wipes (13.27%) were used at similar rates. Additional products included baby or antiseptic wipes (12.12%), liquid or gel sanitizers (10.67%), vaginal sprays (5.48%), and vaginal powders (1.83%). A total of 44.81% of participants reported never using any internal vaginal product, and 29.81% reported no use of external products.

**Table 6:** Types and prevalence of vaginal health and hygiene product use (*N* = 1040)

Product	Ever used (*N* = 1040)	
	Internal *n* (%)	External *n* (%)
Vaginal moisturizers/lubricants	166 (15.96)	141 (13.56)
Vaginal tablets	91 (8.75)	48 (4.62)
Anti-itch creams	180 (17.31)	229 (22.02)
Vaginal wipes	88 (8.46)	138 (13.27)
Vaginal washes/cleansers	397 (38.17)	454 (43.65)
Baby/antiseptic wipes	85 (8.17)	126 (12.12)
Hand/body lotion	105 (10.1)	196 (18.85)
Baby oil	99 (9.52)	153 (14.71)
Vaginal deodorant suppositories	102 (9.81)	54 (5.19)
Liquid/gel sanitizers	101 (9.71)	111 (10.67)
Vaginal sprays	38 (3.65)	57 (5.48)
Vaginal powders	22 (2.12)	19 (1.83)
Shaving cream	81 (7.79)	141 (13.56)
None	466 (44.81)	310 (29.81)

### Association between vaginal hygiene product use and vaginal microbiota knowledge

Unadjusted linear regression analysis examined the association between the use of vaginal hygiene products and participants’ knowledge of the vaginal microbiome (Table 7). Among internal products, only the use of vaginal washes or cleansers was significantly associated with lower knowledge scores (β = − 0.30, 95% CI: − 0.58 to − 0.01, *p* = 0.0403). Other internal products, including vaginal tablets (β = 0.36, *p* = 0.0785), vaginal wipes (β = − 0.32, *p* = 0.1203), and deodorant suppositories (β = − 0.25, *p* = 0.1805), did not show significant associations. Use of internal moisturizers, anti-itch creams, powders, sprays, and other listed products also showed no significant relationship with microbiota knowledge (*p* > 0.05). Similarly, none of the external hygiene products were significantly associated with knowledge scores. External shaving cream use showed a borderline association with higher knowledge (β = 0.28, *p* = 0.0768), while the use of external vaginal washes (β = − 0.17, *p* = 0.1827) and deodorant suppositories (β = − 0.15, *p* = 0.5434) demonstrated negative but non-significant associations. Reporting no use of internal or external hygiene products was also not significantly associated with microbiota knowledge (*p* = 0.2513 and *p* = 0.0914, respectively).

**Table 7:** Unadjusted associations between vaginal hygiene product use and vaginal microbiota knowledge (VMK) score

Practice	Vaginal microbiota knowledge		
	Beta Estimate	95% CI	*p* value
Have you ever used any of the following products in your internal genitals?			
Vaginal moisturizers/lubricants	−0.04	−0.34 to 0.27	0.8144
Vaginal tablets	0.36	−0.04 to 0.75	0.0785
Anti-itch creams	0.02	−0.29 to 0.33	0.904
Vaginal wipes	−0.32	−0.73 to 0.08	0.1203
Vaginal washes/cleansers	−0.30	−0.58 to −0.01	**0.0403** *
Baby/antiseptic wipes	0.13	−0.29 to 0.55	0.5526
Hand/body lotion	−0.05	−0.43 to 0.34	0.8103
Baby oil	0.22	−0.17 to 0.62	0.2709
Vaginal deodorant suppositories	−0.25	−0.62 to 0.12	0.1805
Liquid/gel sanitizers	−0.03	−0.39 to 0.33	0.8715
Vaginal sprays	0.14	−0.44 to 0.72	0.6343
Vaginal powders	0.30	−0.45 to 1.05	0.4299
Shaving cream	0.13	−0.28 to 0.54	0.5272
None	−0.18	−0.49 to 0.13	0.2513
Have you ever used any of the following products in your external genitals?			
Vaginal moisturizers/lubricants	0.01	−0.30 to 0.33	0.926
Vaginal tablets	0.23	−0.29 to 0.75	0.3947
Anti-itch creams	0.13	−0.14 to 0.39	0.3488
Vaginal wipes	−0.05	−0.38 to 0.27	0.7461
Vaginal washes/cleansers	−0.17	−0.41 to 0.08	0.1827
Baby/antiseptic wipes	−0.02	−0.35 to 0.32	0.9195
Hand/body lotion	0.15	−0.13 to 0.44	0.2968
Baby oil	0.11	−0.20 to 0.42	0.4952
Vaginal deodorant suppositories	−0.15	−0.64 to 0.34	0.5434
Liquid/gel sanitizers	0.04	−0.30 to 0.38	0.8145
Vaginal sprays	−0.05	−0.53 to 0.43	0.8452
Vaginal powders	−0.12	−0.94 to 0.70	0.7763
Shaving cream	0.28	−0.030 to 0.59	0.0768
None	−0.25	−0.55 to 0.04	0.0914

Linear regression was used to explore associations between self-reported use of vaginal hygiene products and the VMK score (range: 0–5). Each coefficient (β) reflects the unadjusted association between product use and VMK score.

## Discussion

This cross-sectional online survey aimed to assess Saudi women’s knowledge of the human and vaginal microbiome and to examine its association with vaginal hygiene practices. Overall, our findings indicate moderate awareness of the human microbiome but limited knowledge specific to the vaginal microbiome, particularly regarding beneficial microorganisms and the effects of hygiene practices on microbial balance. While participants demonstrated some awareness of microbial presence in certain body sites, misconceptions about microorganisms being universally harmful were common. In addition, the use of internal and external vaginal hygiene products was widespread, and the use of vaginal washes was inversely associated with vaginal microbiome knowledge, even after adjustment for sociodemographic factors. Together, these findings directly address the study objectives and point to important gaps in vaginal microbiome literacy among women in Saudi Arabia.

Our study found that fewer than half of the participants correctly understood the term “microbiome,” and notably, a substantial majority (85.38%) incorrectly viewed all microorganisms on the human body as harmful. This widespread misunderstanding aligns with previous observations. For instance [36], conducted a cross-sectional survey at a U.S. academic medical center and discovered that only 35% of respondents were familiar with the term “microbiome,” with many perceiving all microbes as harmful. Similarly [37], surveyed individuals across France, Germany, South Korea, and Taiwan, finding that baseline perceptions of microorganisms were slightly negative to neutral. However, after a brief reflection on the role of the microbiome in human health, participants exhibited a modest positive shift in their attitudes toward microorganisms. These findings suggest that public misconceptions about microbial communities are prevalent but can be addressed through targeted educational interventions. Nonetheless, participants in our study exhibited relatively higher awareness of microbial presence in specific anatomical locations, such as the intestinal tract and skin, which may be attributed to the impact of targeted health education focused on those body sites.

Consistent with prior research, our study observed that sociodemographic factors significantly influenced microbiome awareness. Healthcare professionals demonstrated notably higher knowledge scores, highlighting the critical role of professional healthcare training in microbiome literacy [38]. found that among healthcare professionals in Qatar, 95% acknowledged having minimal to no knowledge about the role of gut microbes in health and disease, yet nearly all recognized the importance of considering gut microbiota in treatment plans. Similarly [39], reported that while 94.6% of dental professionals in Saudi Arabia were familiar with the term “microbiome,” only a small fraction understood its diversity and implications for oral and systemic health. These findings underscore the necessity for enhanced microbiome education within healthcare training programs. Furthermore, our study found that participants with only a high school education had significantly lower knowledge than those holding a bachelor’s degree, indicating that formal higher education substantially contributes to microbiome understanding. This aligns with the observations of [40], who noted that adolescents’ knowledge of the microbiome varied with their level of scientific study, suggesting that educational attainment plays a crucial role in microbiome literacy.

Our findings also revealed notable differences in microbiome knowledge based on age, marital status, employment, and geographic region. Participants aged 30–40 years demonstrated significantly higher general microbiome knowledge compared to younger participants, possibly reflecting greater life experience, exposure to healthcare services, or targeted health education. This is consistent with prior studies showing that microbiome knowledge tends to increase with educational level and age-related experience [40]. Regionally, participants from the Northern region reported higher vaginal microbiome knowledge, which may relate to differences in public health programming or healthcare access. Interestingly, retired individuals had higher vaginal microbiome knowledge than students, potentially due to cumulative exposure to healthcare information across the lifespan. Conversely, widowed participants exhibited significantly lower knowledge scores, which may reflect barriers related to aging, social isolation, or limited engagement with health services. These sociodemographic insights reinforce the need for targeted and culturally sensitive educational interventions to address microbiome literacy gaps across different population groups [38, 39].

Specific knowledge regarding the vaginal microbiome was moderate but revealed important gaps. While a majority of participants recognized its protective role and understood that antibiotics and douching can affect microbial balance, only around 20% correctly identified *Lactobacillus* as the dominant genus in a healthy vaginal environment. This reflects persistent uncertainty about microbiome composition, despite global efforts to raise awareness [41, 3].

These findings are consistent with recent work by [42], who found low microbiota awareness among women planning pregnancy, including limited knowledge of the vaginal ecosystem and its relevance to reproductive health. Improving public understanding of the vaginal microbiome is essential, as its disruption can increase susceptibility to conditions such as bacterial vaginosis, urinary tract infections, and adverse pregnancy outcomes. Educational efforts tailored to local cultural norms and product usage behaviors may be more effective in addressing these misconceptions.

In terms of behavioral practices, our study revealed high usage of vaginal hygiene products among Saudi women, consistent with patterns observed internationally. More than half of the participants reported using internal products, and over 70% used external products. Vaginal washes and cleansers were the most commonly used, reflecting similar trends reported by [43] in a national Canadian survey, where a high prevalence of feminine hygiene product use was noted, often without medical guidance.

Importantly, our analysis found a significant negative association between the use of vaginal washes and knowledge of the vaginal microbiome. This finding aligns with previous research linking such practices to disruptions in microbial balance and increased risk of bacterial vaginosis [44] [45]. found that behaviors associated with bacterial vaginosis, such as the use of certain vaginal hygiene products, may increase the risk of developing pelvic inflammatory disease. This highlights how routine hygiene practices could lead to serious reproductive health complications. These practices are often driven by social norms and marketing messages that equate internal cleansing with cleanliness, despite evidence suggesting they may undermine vaginal health.

Although most internal and external vaginal hygiene products were not significantly associated with microbiome knowledge, their widespread use underscores the importance of continued public health monitoring. Interestingly, even participants who reported no use of these products did not show higher knowledge scores, suggesting that non-use alone is not a marker of greater microbiome awareness. These findings highlight the need for broad educational efforts that address misconceptions across the population, including both users and non-users of vaginal hygiene products.

Collectively, our findings underscore the urgent need for targeted microbiome education and public health initiatives in Saudi Arabia. Improving microbiome literacy could help women make more informed decisions about vaginal hygiene practices, reduce the use of potentially harmful products, and support better reproductive health outcomes. In addition to individual education, policy-level action is needed to regulate the marketing of vaginal health products and ensure the dissemination of accurate, evidence-based information. Healthcare professionals should also be equipped to provide microbiome-related guidance as part of routine care, thereby contributing to long-term improvements in women’s health.

This study also addresses a critical gap in the regional literature. As highlighted by [46], microbiome research in Saudi Arabia has primarily focused on the gut, with limited attention to other body sites, including the vaginal microbiome. By examining both awareness levels and related behavioral practices, our study provides foundational data to inform future research, health education strategies, and culturally appropriate interventions in the region.

In conclusion, this study provides an overview of vaginal microbiome knowledge and hygiene practices among women in Saudi Arabia. Despite increased global attention to microbiome science, substantial knowledge gaps remain, particularly in understanding the role of beneficial bacteria and the risks associated with commonly used products. These findings highlight the influence of education, healthcare exposure, and cultural practices on women’s decisions related to vaginal health. Public health initiatives, professional training, and regulatory oversight are essential to promote microbiome-informed behaviors and improve reproductive health outcomes across the region.

Several limitations should be considered. First, the use of an online convenience sampling strategy likely introduced selection bias, resulting in the over-representation of younger women, students, and unmarried participants, as reflected in the sample characteristics. Women who had internet access and were active on social media were more likely to participate. Consequently, the findings cannot be considered nationally representative of all women in Saudi Arabia, particularly older women, those with lower educational attainment, or those living in rural areas with limited internet access. In addition, the cross-sectional design precludes causal inference, and self-reported data may be subject to recall or social desirability bias. Nevertheless, the large sample size allowed exploration of associations between microbiome knowledge and vaginal hygiene practices with reasonable precision within this population.

## Conclusions

This study provides an assessment of vaginal microbiome knowledge and hygiene practices among women in Saudi Arabia. Despite global advances in microbiome science, significant gaps remain in women’s understanding of the role of beneficial bacteria in vaginal health. Only a small proportion of participants correctly identified *Lactobacillus* as the dominant genus in a healthy vaginal environment. While general awareness of microbial presence in the body was moderate, misconceptions about the harmfulness of all microbes were common.

Sociodemographic factors such as education level, employment in the healthcare sector, and geographic region were strongly associated with microbiome knowledge. Moreover, a high prevalence of intimate hygiene product use was reported, particularly vaginal washes and anti-itch creams. Notably, internal vaginal washes were significantly associated with lower levels of microbiome knowledge, suggesting a gap between behavior and understanding of potential health impacts.

These findings underscore the urgent need for public health strategies and targeted educational interventions to improve microbiome literacy. Empowering women with accurate, accessible information is critical to supporting microbiota-friendly hygiene behaviors and improving reproductive health outcomes in the region.

## Supplementary Information

Supplementary Material 1.

## Data Availability

The datasets generated and/or analyzed during the current study are available from the corresponding author on reasonable request.

## Abbreviations

BV: Bacterial Vaginosis
CI: Confidence Interval
IRB: Institutional Review Board
UTIs: Urinary Tract Infections
