# Supplementary material for: Vaginal microbiome knowledge and hygiene practices among women in Saudi Arabia: a cross-sectional study
Source: BMC Public Health. 2025 Dec 28;26:393. doi: 10.1186/s12889-025-26075-9 (PMC12853612; doi:10.1186/s12889-025-26075-9)
Supplement: Supplementary file 1 — Supplementary Material 1. [file 12889_2025_26075_MOESM1_ESM.docx]

**Vaginal Microbiome Knowledge and Hygiene Practices Among Women in Saudi Arabia – Study Questionnaire (English Version)**

**Section 1: Sociodemographic Information**

1. Age
   - < 20 years
   - 20–30 years
   - 30–40 years
   - 40–50 years
   - 50 years
2. Marital status
   - Single
   - Married
   - Divorced
   - Widowed
3. Educational level
   - Elementary school
   - High school
   - Bachelor’s degree
   - Postgraduate degree
4. Employment status
   - Student
   - Employed full-time
   - Employed part-time
   - Self-employed
   - Unemployed
   - Retired
5. Do you work in the healthcare sector?
   - Yes
   - No
6. Region of residence in Saudi Arabia
   - Central
   - Eastern
   - Western
   - Northern
   - Southern

**Section 2: Knowledge of the Human Microbiome**

*(True / False / I don’t know)*

1. The term “microbiome” refers to all microorganisms living in the human body.
2. All microorganisms found on the human body are harmful.
3. Microbiome composition is similar for all people.
4. There are microorganisms living naturally in the intestinal tract.
5. There are microorganisms living naturally in the respiratory tract.
6. There are microorganisms living naturally on the skin.
7. There are microorganisms living naturally in the vagina.
8. Misuse of antibiotics can negatively affect the microbiota.

**Section 3: Knowledge of the Vaginal Microbiome**

*(True / False / I don’t know)*

1. The vaginal microbiome plays a crucial role in maintaining vaginal health and protecting against pathogenic microorganisms.
2. The vaginal microbiome is dynamic and dominated by *Lactobacillus* species in health.
3. The composition of the vaginal microbiota can change throughout a woman’s menstrual cycle.
4. Use of antibiotics can cause temporary changes in the vaginal microbiota.
5. Vaginal douching can disrupt the vaginal microbiota by altering the microbial community.

**Section 4: Vaginal Hygiene and Health Practices**

*(Select all that apply if applicable)*

**Internal vaginal products:**

1. Vaginal moisturizers/lubricants
2. Vaginal tablets
3. Anti-itch creams
4. Vaginal wipes
5. Vaginal washes/cleansers
6. Baby or antiseptic wipes
7. Hand or body lotion
8. Baby oil
9. Vaginal deodorant suppositories
10. Liquid or gel sanitizers
11. Vaginal sprays
12. Vaginal powders
13. Shaving cream
14. None

**External vaginal products:**

1. Vaginal moisturizers/lubricants
2. Vaginal tablets
3. Anti-itch creams
4. Vaginal wipes
5. Vaginal washes/cleansers
6. Baby or antiseptic wipes
7. Hand or body lotion
8. Baby oil
9. Vaginal deodorant suppositories
10. Liquid or gel sanitizers
11. Vaginal sprays
12. Vaginal powders
13. Shaving cream
14. None

**Section 5: Sources of Information**

1. From where do you usually obtain information about feminine hygiene and the vaginal microbiome?
   - Internet/social media
   - Friends or family
   - Healthcare professionals
   - University courses or lectures
   - Awareness campaigns
   - Other (please specify)
